# Evaluation of Genotoxic Effects of N-Methyl-N-Nitroso-Urea and Etoposide on the Differentiation Potential of MSCs from Umbilical Cord Blood and Bone Marrow

**DOI:** 10.3390/cells13242134

**Published:** 2024-12-23

**Authors:** Meryem Ouzin, Sebastian Wesselborg, Gerhard Fritz, Gesine Kogler

**Affiliations:** 1 Institute for Transplantation Diagnostics and Cell Therapeutics, University Hospital, Heinrich Heine University Düsseldorf, Moorenstraße 5, 40225 Düsseldorf, Germany; gesine.koegler@med.uni-duesseldorf.de; 2 Institute for Molecular Medicine I, University Hospital, Heinrich Heine University Düsseldorf, Universitätsstraße 1, 40225 Düsseldorf, Germany; sebastian.wesselborg@uni-duesseldorf.de; 3 Institute of Toxicology, University Hospital, Heinrich Heine University Düsseldorf, Universitätsstraße 1, 40225 Düsseldorf, Germany; fritz@uni-duesseldorf.de

**Keywords:** mesenchymal stromal cells, osteogenesis, adipogenesis, chondrogenesis, nitrosamines, etoposide

## Abstract

The present study investigates the influence of nitrosamines and etoposide on mesenchymal stromal cells (MSCs) in a differentiation state- and biological age-dependent manner. The genotoxic effects of the agents on both neonatal and adult stem cell populations after treatment, before, or during the course of differentiation, and the sensitivity of the different MSC types to different concentrations of MNU or etoposide were assessed. Hereby, the multipotent differentiation capacity of MSCs into osteoblasts, adipocytes, and chondrocytes was analyzed. Our findings reveal that while all cell types exhibit DNA damage upon exposure, neonatal CB-USSCs demonstrate enhanced resistance to genotoxic damage compared with their adult counterparts. Moreover, the osteogenic differentiation of MSCs was more susceptible to genotoxic damage, whereas the adipogenic and chondrogenic differentiation potentials did not show any significant changes upon treatment with genotoxin. Furthermore, we emphasize the cell-specific variability in responses to genotoxic damage and the differences in sensitivity and reaction across different cell types, thus advocating the consideration of these variabilities during drug testing and developmental biological research.

## 1. Introduction

Mesenchymal stromal cells (MSCs) are a heterogeneous population that was originally identified in the bone marrow [1,2] but has also been discovered in various other tissues, such as adipose tissue, umbilical cord blood, or placenta, thus offering unique advantages regarding accessibility for clinical applications [3,4,5]. Due to their low expression of the Major Histocompatibility Complex (MHC) I and lack of the MHC II, MSCs are recognized for their immunomodulatory properties on T-cell alloreactive responses and proliferation but also for their ability to secrete bioactive molecules such as cytokines, chemokines and growth factors that promote tissue repair and regeneration [6]. This versatility makes them a focal point in regenerative medicine and tissue engineering, offering promising approaches for the treatment of a wide range of diseases and injuries.

Beyond these immunomodulatory capacities, MSCs are also known for their multipotent differentiation capacity, which allows them to differentiate into a variety of cell types, including osteoblasts, chondrocytes, and adipocytes. All these properties make them ideal candidates for cell-based therapies aiming for tissue repair or replacement in conditions such as osteoarthritis, cardiovascular diseases, and neurodegenerative disorders. This ability is driven by complex signaling pathways and micro-environmental cues, highlighting the importance of understanding these mechanisms to harness the full therapeutic potential of MSCs. This knowledge can furthermore be exploited in the field of toxicological risk assessment. The use of MSCs for drug testing represents a significant advancement in pharmacological and toxicological research, offering a reliable and human-relevant model for evaluating drug toxicity. The multilineage differentiation potential of MSCs allows for comprehensive testing across different tissue types, thus providing more accurate predictions of a drug impact, enhances the predictive power of preclinical studies and, moreover, reduces the reliance on animal models, which often fail to reflect human physiology.

MSCs are integral components of the bone marrow niche and thus play a crucial role in supporting hematopoiesis by providing essential signals and a supportive microenvironment for the proliferation and differentiation of hematopoietic stem cells (HSCs). This unique interaction underscores their importance in drug toxicity testing, as they can influence the hematopoietic response to chemical substances. Thus, evaluating drug effects on MSCs is essential for understanding potential toxicities and side effects on the bone marrow and overall hematopoietic function, thereby enhancing the safety and efficacy profiles of new therapeutic agents.

Biological age is a crucial factor in drug testing since it influences how cells and tissues respond to chemical compounds. Cells from younger sources with a lower biological age typically exhibit higher proliferative capacity, robust regenerative properties, and predictable responses to drugs for an accurate assessment of drug efficacy and toxicity [7,8]. Conversely, cells with a higher biological age often have accumulated genetic and epigenetic changes, reduced regenerative capacity, and altered metabolic profiles that can affect drug metabolism and efficacy [9]. Incorporating the concept of biological age into drug testing helps to ensure that the models used closely mimic the intended patient populations, thus enhancing the relevance and reliability of preclinical studies. This approach can lead to better predictions of clinical outcomes, reduce the incidence of adverse effects, and improve the overall success rate of new drug development. The ability to isolate MSCs from various sources provides a unique opportunity to analyze age-dependent reactions to toxins. This versatility allows the comparison of MSC responses across different age groups, from neonatal to adult, thereby gaining insights into how age-related cellular and molecular changes influence responses to drugs. This is also crucial for developing age-specific drug safety profiles, optimizing therapeutic strategies, and minimizing adverse effects in different patient populations, ultimately leading to more personalized and effective treatments.

The biological age difference between cord blood MSCs (CB-MSCs), cord blood unrestricted somatic stem cells (CB-USSCs), and bone marrow MSCs (BM-MSCs) is a critical factor influencing their therapeutic potential and functionality. MSCs derived from cord blood (CB-MSCs and CB-USSCs) are derived from a perinatal source, making them biologically younger and more primitive compared with BM-MSCs, which are obtained from adult tissues. This is associated with a higher proliferative capacity, greater telomerase activity, and enhanced differentiation potential [10]. Additionally, CB-USSCs and -MSCs exhibit lower immunogenicity and a greater ability to modulate immune responses, which can be advantageous in clinical applications. In contrast, adult BM-MSCs, although well studied and widely used, may have reduced regenerative abilities due to the aging process and accumulation of environmental stress-induced damage. Understanding these differences is crucial for optimizing the selection of MSC sources for specific therapeutic and risk assessment purposes.

Nitrosamines are a class of chemical compounds typically formed through the reaction of nitrites and secondary amines, often found in processed meats, tobacco smoke, and certain industrial settings. These compounds are potent carcinogens, known for their ability to induce DNA damage through alkylation, leading to mutations that can initiate cancer development [11,12]. Upon metabolic activation, nitrosamines produce reactive intermediates that form adducts with DNA, resulting in mutagenesis and potentially triggering a cascade of cellular malfunctions. Chronic exposure to nitrosamines has been linked to various cancers, including those of the liver, esophagus, and stomach, highlighting the importance of monitoring and limiting exposure to these toxic substances to mitigate their health risks [13,14,15].

Etoposide is a chemotherapeutic agent commonly used to treat various cancers, including testicular cancer, lung cancer, and lymphoma. It functions primarily by inhibiting the enzyme topoisomerase II, which is crucial for DNA replication and transcription. By stabilizing the DNA-topoisomerase II complex, etoposide induces DNA double-strand breaks (DSBs) and prevents their re-ligation, leading to DSB accumulation. This disruption in DNA integrity triggers cell cycle arrest and apoptosis in rapidly dividing cancer cells [16]. However, the toxic effects of etoposide also extend to normal, healthy cells, particularly those with high proliferation rates, such as bone marrow cells, leading to side effects like myelosuppression, increased risk of secondary hematological malignancies, and other systemic toxicities [17,18]. The ability of etoposide to cause DNA damage underpins its effectiveness as a cancer treatment but also necessitates careful management to mitigate adverse effects on healthy tissue.

Drug and chemical testing on both target cancer cells and their surrounding non-cancer cells is crucial to comprehensively assess the safety and efficacy of new therapies. While specifically targeting diseased cells is essential for therapeutic efficacy, the impact on neighboring healthy cells must also be evaluated to predict potential side effects and systemic toxicity. Understanding how chemicals affect the microenvironment, including supportive stromal cells, immune cells, and endothelial cells, helps to identify unintended harmful effects that could compromise patient health. This holistic approach ensures that treatments are not only effective against the intended targets but also safe for the overall cellular milieu, leading to more reliable and effective therapeutic strategies with minimized adverse effects. Furthermore, this study emphasized the need to evaluate not only direct cytotoxicity on healthy tissues but also the influence of genotoxins both on the differentiation capacity of tissue stem cells and on the responses to genotoxic damage at different stages of cellular development. By conducting analyses at various time points following the induction of differentiation, we sought to evaluate the effects of genotoxin treatment on the initiation and progression of MSC differentiation. Additionally, this approach allows comparing the susceptibility of undifferentiated with differentiated MSCs. This dual focus provides insights into the potential risks posed by genotoxins in terms of regenerative processes and mature tissue functionality.

## 2. Materials and Methods

### 2.1. Generation and Expansion of CB-MSCs and CB-USSCs

Cord blood was collected from umbilical cord vein with informed consent of the mother. Briefly, mononuclear cells (MNC) were obtained by Ficoll^®^ (Biochrom, Berlin, Germany, density 1.077 g/cm^3^) gradient separation following lysis of RBCs by ammonium chloride. A total of 5–7 × 10^6^ CB-MNC/mL was cultured in low-glucose Dulbecco’s modified Eagle’s medium (DMEM) (Lonza, Basel, Switzerland) with 30% fetal calf serum (FCS) (Perbio, Bonn, Germany), 10^–7^ M dexamethasone (Sigma-Aldrich, St-Louis, MO, USA), and penicillin/streptomycin (Lonza) until detection of adherent growing colonies. Cells were expanded without dexamethasone in a closed system applying cell stacks (Corning, New York, NY, USA), incubated at 37 °C in 5% CO_2_ in a humidified atmosphere. After reaching 80% confluency, cells were detached with 0.25% trypsin (Lonza) and re-plated 1:3. CB-USSCs were distinguished from CB-MSCs by analysis of their HOX gene expression pattern by PCR [19]. CB-USSCs lack most HOX gene clusters. The used CB-MSC and CB-USSC cell lines are listed in Table S1.

### 2.2. Generation of BM-MSCs

Bone marrow from healthy adult donors was directly plated for the generation of MSCs in DMEM low glucose with 30% FCS and PS until adherent colonies appeared. Cells were expanded without dexamethasone in a closed system applying cell stacks (Corning), incubated at 37 °C in 5% CO_2_ in a humidified atmosphere. After reaching 80% confluency, cells were detached with 0.25% trypsin (Lonza) and re-plated 1:3. The used BM-MSC cell lines are listed in Table S1.

### 2.3. Generation and Expansion of iPSCs

Clinical-grade iPSCs were generated from selected cord blood units based on informed re-consenting of the donors as described by Terheyden-Keighley et al. [20].

### 2.4. MNU Treatment

Prior to treatment of cultivated cells with MNU (MedChemExpress, Monmouth Junction, NJ, USA), cells were washed twice with PBS (Lonza) and incubated for 7 min at 37 °C. After removal of PBS, different concentrations of MNU (stock solution: 1 M in DMSO) were added to the cells. After 1 h treatment, MNU was removed prior to further cultivation or differentiation.

### 2.5. Etoposide Treatment

Treatment with etoposide (TCI Europe N. V., Zwijndrecht, Belgium) was conducted for 24 h in culture medium. After treatment with different etoposide concentrations (stock solution: 100 µM in DMSO), cells were washed with PBS prior to further cultivation or differentiation.

### 2.6. Cell Viability Assay

Resazurin staining was used to determine cell viability 4 days after cytotoxic treatment (1 h MNU or 24 h Etoposide). Resazurin is reduced to fluorescent resorufin by metabolically active cells. Cells were cultured in 96-well plates, treated with resazurin solution after 4 days, and incubated for 2–4 h. The fluorescence intensity, which is proportional to the number of living cells, was measured using a fluorescence microplate reader (excitation wavelength: 560 nm; emission wavelength: 590 nm).

### 2.7. Assessment of MSC Growth

To evaluate the cumulative population doublings (CPD), the following formula was applied: PD = [log(n_1_/n_0_)]/log_2_ CPD = ΣPD, where n_1_ is the number of harvested cells, and n_0_ is the number of plated cells. Cells were treated 1 h with MNU or 24 h with etoposide, and proliferation measurements were carried out for several passages.

### 2.8. In Vitro Osteogenic Differentiation and Alizarin Staining

In vitro osteogenic differentiation was conducted as described by Liedtke et al. [21]. Uninduced cells were used as a negative control. After culturing for 14 days, the cells were fixed with 4% paraformaldehyde at 4 °C for 20 min. Next, the fixed cells were stained with 40 mM Alizarin Red S (Sigma-Aldrich) for 30 min at room temperature and observed under an inverted microscope.

For quantification of calcium deposition, 800 µL 10% (*v*/*v*) acetic acid was added to the cells, incubated at room temperature for 30 min with shaking until scaffolds detached, and collected. After vortexing for 30 s, the samples were heated to exactly 85 °C for 10 min, transferred to ice for 5 min, and centrifuged at 345× *g* for 15 min. The supernatant was removed into a collecting tube, and 200 µL of 10% (*v*/*v*) ammonium hydroxide was added to neutralize the acid. OD was measured in triplicates at 405 nm on a 96-well plate.

### 2.9. In Vitro Adipogenic Differentiation and Oil Red O Staining

In vitro adipogenic differentiation was conducted as described by Liedtke et al. [21]. Uninduced MSCs were used as a negative control. The cells were maintained for 21 days and then fixed with 37% formaldehyde at room temperature for 10 min. Finally, the cells were examined under an inverted microscope after staining with 0.3% Oil Red O (Sigma-Aldrich) in 60% isopropanol for 20 min.

For quantification, Oil Red O was extracted by adding 500 µL isopropanol. OD was measured in triplicated at 500 nm on a 96-well-plate.

### 2.10. In Vitro Chondrogenic Differentiation and Safranin O Staining

In vitro adipogenic differentiation was conducted as described by Liedtke et al. [21]. The spheroidal chondrogenic pellets were embedded in Tissue Freezing Medium (Leica, Wetzlar, Germany) and frozen at −80 °C before cutting them into sections of 6 µm using a cryotome (Leica). The areas and diameters of the pellets were measured on days 7, 14, and 21 of differentiation using AVISO CellCelector analySIS image software (Version 2.7). For each time point, a minimum of n = 7 pellets were measured, and the arithmetic mean and standard deviation (SD) were calculated.

### 2.11. Total RNA Extraction and Reverse Transcription

Total RNA was extracted from the cell pellets in a 30 µL volume applying the RNeasy Kit (Qiagen, Hilden, Germany) according to the manufacturer’s instructions, including the optional 15 min DNase digest. RNA from differentiated cells was isolated using the TRI Reagent^®^ RNA Isolation reagent (Sigma-Aldrich) following a standard protocol of a phenol–chloroform extraction. Determination of RNA concentrations and purity was carried out using a Nanodrop device (NanoDropTechnologies, Wilmington, NC, USA). Reverse transcription was applied using the first-strand cDNA synthesis kit (Invitrogen, Waltham, MA, USA) and oligo(dT)_20_ primer (Thermo Fisher Scientific, Waltham, MA, USA) following the manufacturer’s instructions. A total of 1 µg of total RNA was converted into first-strand cDNA in a 20 µL reaction.

### 2.12. Quantitative PCR Analysis (RT-qPCR)

RT-qPCR analysis was carried out with intron-spanning primers specific to each gene (Thermo Fisher Scientific). The respective primer sequences are given in Table S2. RPL13a was used as a reference gene. A total of 50 ng of cDNA was applied for RT-qPCR in a total volume of 25 µL containing Sybr Green PCR Mastermix (Thermo Fisher Scientific), 0.2 µM of primer (forward + reverse), and distilled water (10 min/95 °C–15 s/95 °C–1 min/60 °C for 40 cycles).

To analyze the comparative CT experiments, Step One Software v.1.5.1 was used. Relative changes in gene expression were calculated by applying the comparative ∆∆CT method. Differential gene expression was calculated by the formula 2^−∆∆CT^ normalized to untreated cells. Fold changes < 1 were transformed by the formula −1/2^−∆∆CT^ in the case of downregulated genes and plotted together with positive fold changes and upregulated genes, respectively.

### 2.13. Alkaline Comet Assay

A total of 1–2 × 10^6^ cells were washed with PBS, pelleted, and stored on ice or at −20 °C. The cell number used for the assay is fixed upon all samples to be compared. A total of 10 µL of the cell suspension was added to 120 µL LMP-Agarose (37 °C), placed on an object slide, and covered with a coverslip, which was then cooled for 5 min at 4 °C. Next, the coverslip was carefully laterally removed and incubated for 1 h in precooled lysis buffer (2.5 M NaCl, 100 mM EDTA, 10 mM Tris, 1% lauroylsarcosine, Triton X-100, DMSO) at 4 °C. The slides were removed from the lysis buffer, allowed to drain, and placed into an electrophoresis chamber before overlayering (2–3 mm) with precooled electrophoresis buffer. After an incubation step of 25 min for alkaline denaturation of DNA at 4 °C, electrophoresis was carried out (25 min, 25 V, and 300 mA) on ice. Subsequently, the slides were removed from the chamber, allowed to drain, and three times overlayered with neutralization buffer (400 mM Tris) for 5 min each. Next, the slides were immersed in ddH_2_O, followed by 5 min incubation in 80–100% ethanol. Next, the slides were diagonally tilted and dried overnight. For imaging, 50 µL PI solution was added onto the slide and covered with a coverslip. A minimum of 50 comets per condition were counted.

### 2.14. Statistical Analysis

Statistical analysis was performed using one-way analysis of variance (ANOVA) to evaluate the differences between groups in response to nitrosamines and etoposide exposure. Quantitative data are presented as means ± SD. Differences were considered significant at *p* ≤ 0.05. * denotes *p* ≤ 0.05, ** denotes *p* ≤ 0.01, *** denotes *p* ≤ 0.001, and **** denotes *p* ≤ 0.0001.

## 3. Results

### 3.1. Determination of Sublethal Treatment Doses of MNU and Etoposide

**Figure 1:**
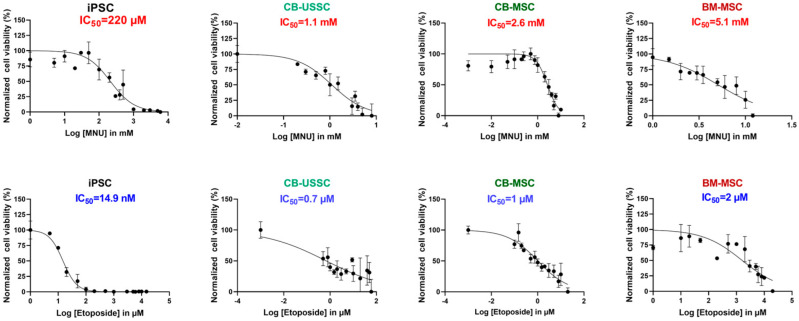
Cell viability and corresponding IC_50_ values after MNU or etoposide treatment measured by resazurin reduction assay. Representative data from an MSC from each source and an iPSC cell line is shown (Mean ± SD from N = 2, n = 3). Abbreviations: CB, cord blood; BM, bone marrow; iPSC, induced pluripotent stem cell; MNU, N-methyl-N-nitroso-urea; USSC; unrestricted somatic stem cells.

Based on the resazurin reduction assay, doses ranging from 1 to 5 mM (for MNU treatment) and 1 to 10 µM (for etoposide treatment) for CB-USSC, CB-MSC, and BM-MSC cell lines were used for further experiments (Figure 1). IPSCs showed a higher sensitivity to both genotoxic agents compared with MSCs, with an IC_50_ of 220 µM for MNU and 14.9 nM for etoposide. When compared with neonatal MSC cell lines, iPSCs showed a lower expression of DNA damage repair (DDR) genes and DDR-related factors (Figure S1). This comes in accordance with their higher vulnerability to genotoxin-induced damage.

### 3.2. Effects of DNA Damage Resulting from MNU or Etoposide Treatment on MSC Morphology and Proliferation Kinetics

**Figure 2:**
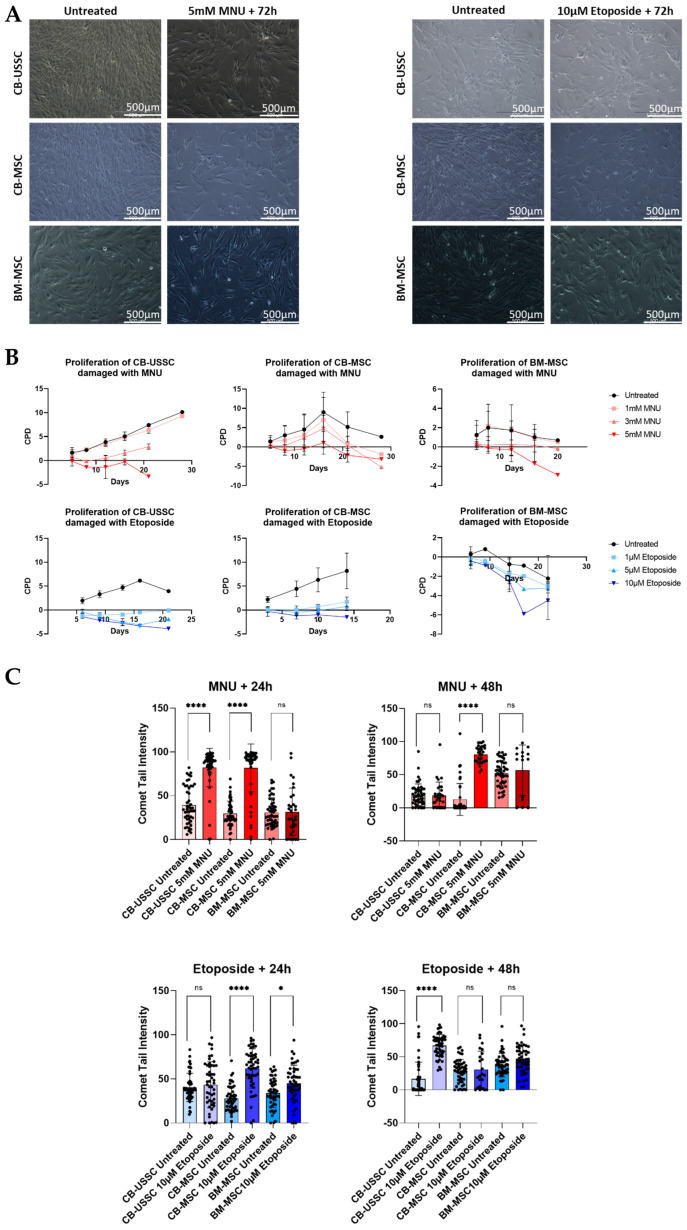
(**A**) Morphological changes in MSCs from different sources 72 h after treatment with 5 mM MNU for 1 h or 10 µM etoposide for 24 h in comparison with the untreated cells. Morphology was representatively analyzed 72 h after 5 mM MNU or 10 µM etoposide treatment. Scale bars = 500 µm. (**B**) Dose-dependent impact of genotoxic stress induced by MNU or etoposide on proliferation kinetics of CB-USSC, CB-MSC, and BM-MSC. To determine long-term growth kinetics, cells were exposed to different doses of MNU (untreated, 1 mM, 3 mM, and 5 mM) or etoposide (untreated, 1 µM, 5 µM, 10 µM), and cell numbers were counted after each passage. CPDs are shown (Mean ± SD from N = 3). (**C**) Analysis of DNA damage in MSCs after MNU or etoposide treatment by alkaline comet assay. For each condition, the intensity of 50 comets was assessed. CB-USSC2, CB-USSC3, CB-USSC4, CB-MSC1, CB-MSC2, CB-MSC3, CB-MSC4, BM-MSC2, BM-MSC3. Quantitative data are presented as means ± SD. * denotes *p* ≤ 0.05 and **** denotes *p* ≤ 0.0001. Abbreviations: BM, bone marrow; CB, cord blood; CPD, cumulative population doublings; MNU, N-methyl-N-nitroso-urea; ns, no significance; USSC; unrestricted somatic stem cells.

MSCs from all sources showed an impaired morphology after treatment with 5 mM MNU or 10 µM etoposide (Figure 2A). The untreated cells have a more elongated and organized morphology, whereas the treated cells are less compact, indicating the effect of the genotoxin treatment on cell proliferation already 72 h after treatment independently of the biological age. Additionally, MNU treatment has a notable decrease in MSC proliferation kinetics, shown by the CPDs traced after treatment and subsequent culture over the course of 20 to 30 days (Figure 2B). CB-USSCs treated with 3 mM and 5 mM MNU only reached a CPD of 2.9 and −3.3, respectively, after 20 days of cultivation. Expansion after 20 days was not possible due to too low cell numbers. CB-MSCs treated with 3 mM MNU reached a CPD of −5.16 after 28 days of culture, whereas treatment of BM-MSCs with 5 mM MNU showed a CPD of −3.18 already after 20 days of cultivation. Treatment with low etoposide concentrations led to a senescent state in CB-USSC and -MSC. Again, adult BM-MSCs show a highly impaired proliferation compared with neonatal MSCs similar to MNU treatment. Treatment of neonatal cells with 10 µM etoposide led to negative CPD values already after the first passaging. Comet assay analysis revealed high amounts of DSBs 24 h after MNU treatment and 48 h after etoposide treatment, especially in CB-USSCs and CB-MSCs (Figure 2C).

### 3.3. Effects of DNA Damage on the Multilineage Differentiation Potential of MSCs

#### 3.3.1. Effects of MNU Treatment on Day 0

- *Osteogenic Differentiation*

In order to determine the effect of MNU treatment on the osteogenic differentiation potential of MSCs, cells were damaged at d0 before osteogenesis induction and subsequent culture until the final readout on d14. Hereby, CB-USSC, CB-MSC, and BM-MSC were compared. The calcification level, which is an indicator of the osteogenic capacity, was assessed by Alizarin staining on d14.

**Figure 3:**
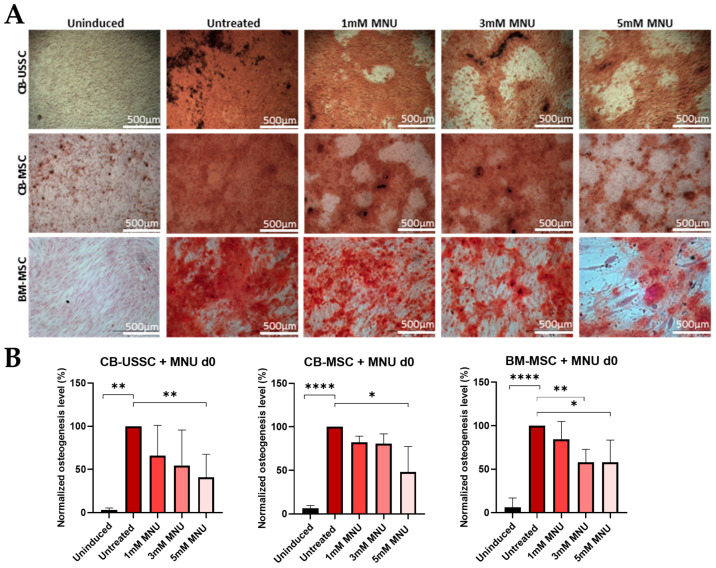
(**A**) Osteogenic differentiation of MSCs after MNU treatment on d0. Representative data (CB-USSC3-CB-MSC3-BM-MSC1) after Alizarin Red S staining on d14 of osteogenic differentiation is shown. Scale bars = 500 µm. (**B**) Osteogenic differentiation of MSCs after 1 h MNU treatment with different doses (untreated, 1 mM, 3 mM, and 5 mM) on d0. Quantitative determination of Alizarin Red S staining on d14 of osteogenic differentiation is shown. Quantitative data are presented as means ± SD of N = 3, n = 3. Differences were considered significant at *p* ≤ 0.05. * denotes *p* ≤ 0.05, ** denotes *p* ≤ 0.01 and **** denotes *p* ≤ 0.0001. Abbreviations: BM, bone marrow; CB, cord blood; d, day; MNU, N-methyl-N-nitroso-urea; USSC; unrestricted somatic stem cells.

MSCs from all sources showed a decreased osteogenic differentiation potential after treatment with MNU on d0 before induction of differentiation (Figure 3). A more than 2-fold decrease in calcification was traced by Alizarin staining after treatment with 5 mM MNU before induction of osteogenic differentiation. Expression levels of osteogenic genes were analyzed at d7 and d14 after induction of osteogenic differentiation. Runt-related transcription factor 2 (RUNX2), which is considered the master regulator of osteogenic differentiation and the stimulator of Osterix (OSX) expression [22], did not show any significant downregulation in the treated samples on both d7 and 14, whereas OSX levels at d14 were downregulated after treatment with high MNU doses in both CB-USSCs and CB-MSCs to reach levels comparable to the uninduced negative control (Figure S2). BM-MSCs first showed an upregulation of OSX levels after treatment with 1 mM or 3 mM MNU and a downregulation in the case of treatment with 5 mM MNU.

- *Adipogenic Differentiation*

**Figure 4:**
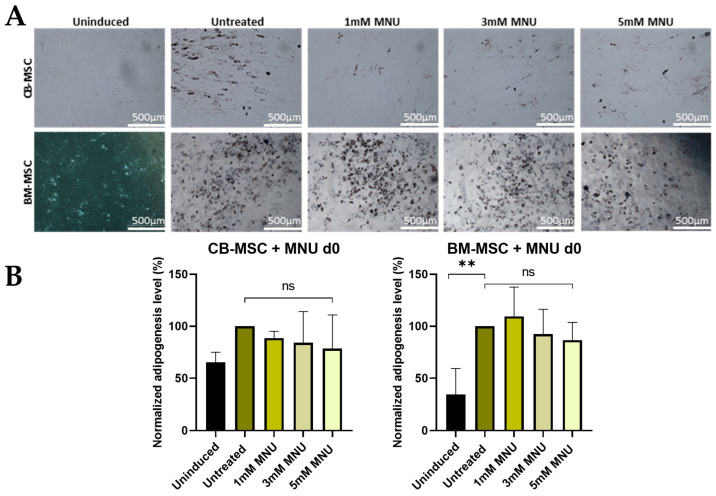
(**A**) Adipogenic differentiation of MSCs after MNU treatment on d0. Representative data (CB-MSC3-BM-MSC4) after Oil Red O staining on d21 of adipogenic differentiation is shown. The induced samples successfully underwent adipogenesis, while the uninduced sample was devoid of characteristic lipid droplet formation after 21 days of differentiation. Scale bars = 500 µm. (**B**) Adipogenic differentiation of MSCs after 1 h MNU treatment with different doses (untreated, 1 mM, 3 mM, and 5 mM) on d0. Quantitative determination of Oil Red O staining on d21 of adipogenic differentiation is shown. Quantitative data are presented as means ± SD of N = 3, n = 3. Differences were considered significant at *p* ≤ 0.05. ** denotes *p* ≤ 0.01, and ns denotes non-significant. Abbreviations: BM, bone marrow; CB, cord blood; d; day; MNU, N-methyl-N-nitroso-urea.

The low amount of characteristic lipid droplets formed during the adipogenic formation of BM–MSCs may be related to the possibly high passage number used for adipogenic differentiation. In contrast to osteogenic differentiation, MNU treatment on d0 had no effect on adipogenic differentiation of CB- and BM-MSCs (Figure 4). CB-USSCs were not assessed since they lack the ability to differentiate into adipocytes [23].

Late adipogenic genes CCAAT/enhancer-binding-proteins (CEBPα and CEBPβ), which play a role in terminal adipocyte differentiation and maturation, showed a dose-dependent increase in MNU-treated cells, whereas the peroxisome proliferator-activated receptor γ (PPARγ), which is considered as the key regulator of adipogenic differentiation, was not significantly dysregulated in treated samples (Figure S3).

- *Chondrogenic Differentiation*

**Figure 5:**
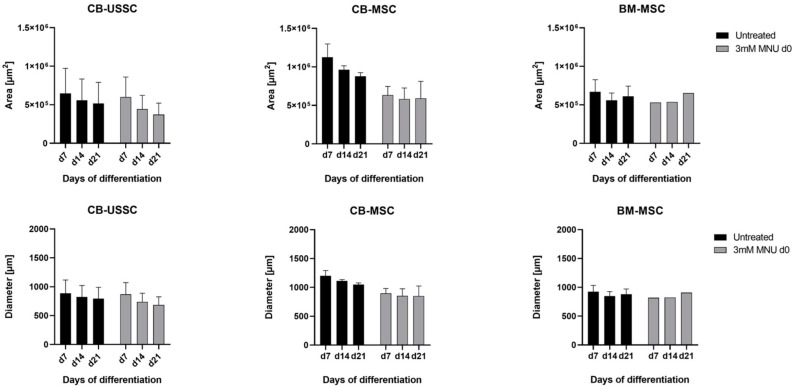
Chondrogenic differentiation of MSCs after 1 h MNU treatment on d0 of differentiation. Areas and diameters of the chondrogenic pellets were measured on d7, d14, and d21 of chondrogenic differentiation (Mean + SD N = 3, n = 3). Abbreviations: BM, bone marrow; CB, cord blood; d; day; MNU, N-methyl-N-nitroso-urea; USSC; unrestricted somatic stem cells.

The effects of 3 mM MNU treatment on the chondrogenic differentiation, in particular, pellet area and pellet diameter of CB-USSC, CB-MSC, and BM-MSC over a period of 7, 14, and 21 days, were assessed. In CB-USSCs, both untreated and MNU-treated conditions showed no significant difference in pellet size over the course of differentiation, indicating no impact of MNU on chondrogenic differentiation (Figure 5). Untreated CB-MSCs showed higher condensation levels compared with the MNU-treated samples, which showed no changes in size over the course of differentiation. However, untreated BM-MSCs exhibit a decreased condensation level compared with the CB-derived cell types. This baseline variation complicates the interpretation of MNU-related effects in BM-MSCs.

#### 3.3.2. Effects of Etoposide Treatment on Day 0

- *Osteogenic Differentiation*

**Figure 6:**
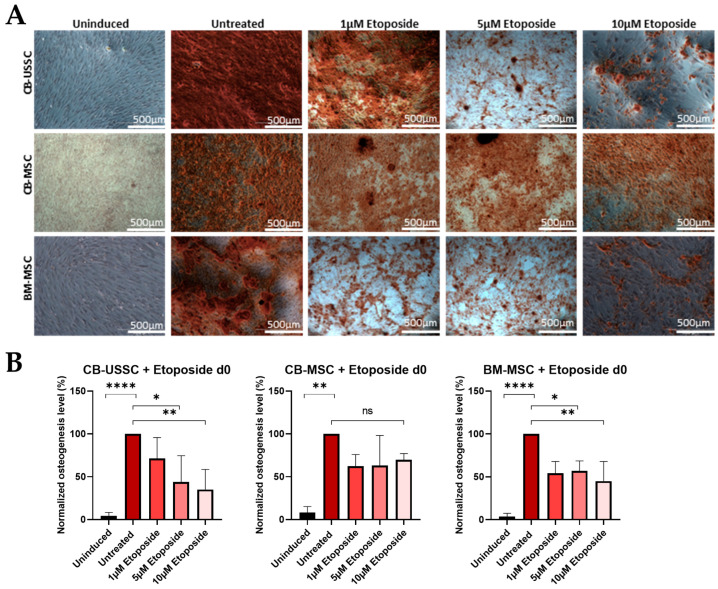
(**A**) Osteogenic differentiation of MSCs after 24 h etoposide treatment on d0. Representative data (CB-USSC4-CB-MSC2- BM-MSC1) after Alizarin Red S staining on d14 of osteogenic differentiation is shown. Scale bars = 500 µm. (**B**) Osteogenic differentiation of MSCs after 24 h etoposide treatment with different doses (untreated, 1 µM, 5 µM, and 10 µM) on d0. Quantitative determination of Alizarin Red S staining on d14 of osteogenic differentiation is shown. Quantitative data are presented as means ± SD of N = 3, n = 3. Differences were considered significant at *p* ≤ 0.05. * denotes *p* ≤ 0.05, ** denotes *p* ≤ 0.01, **** denotes *p* ≤ 0.0001 and ns denotes non-significant. Abbreviations: BM, bone marrow; CB, cord blood; USSC, unrestricted somatic stem cells.

Similarly to MNU treatment on d0, etoposide treatment on d0 led to an impaired osteogenic differentiation potential of CB-USSCs and BM-MSCs but had no significant impact on the osteogenic differentiation potential of CB-MSCs (Figure 6).

Etoposide treatment on d0 led to a downregulation of RUNX2 and OSX expression on d14, especially with high genotoxin doses, in contrast to MNU treatment on d0 (Figure S4).

- *Adipogenic Differentiation*

**Figure 7:**
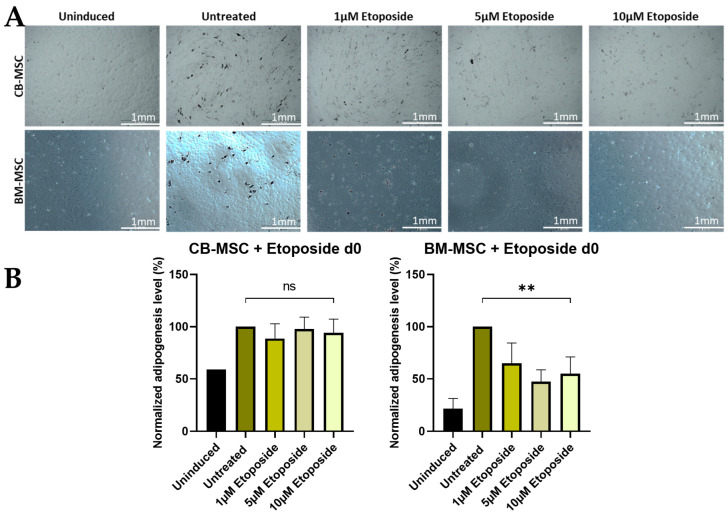
(**A**) Adipogenic differentiation of MSCs after 24 h etoposide treatment on d0. Representative data (CB-MSC2-BM-MSC2) after Oil Red O staining on d21 of adipogenic differentiation is shown. The induced samples successfully underwent adipogenesis, while the uninduced sample was devoid of characteristic lipid droplet formation after 21 days of differentiation. Scale bars = 1 mm. (**B**) Adipogenic differentiation of MSCs after 24 h etoposide treatment with different doses (untreated, 1 µM, 5 µM, and 10 µM) on d0. Quantitative determination of Oil Red O staining on d21 of adipogenic differentiation is shown. Quantitative data are presented as means ± SD of N = 3, n = 3. Differences were considered significant at *p* ≤ 0.05. ** denotes *p* ≤ 0.01, and ns denotes non-significant. Abbreviations: BM, bone marrow; CB, cord blood.

The results indicate that etoposide treatment on d0 has differing effects on the outcome of CB-MSC and BM-MSC adipogenic differentiation. CB-MSCs showed no significant difference in adipogenic potential following MNU treatment, whereas BM-MSCs exhibited a reduced adipogenic potential in response to MNU. Notably, even under untreated conditions, the adipogenic potential of BM-MSCs was inherently lower than that of CB-MSCs, suggesting an intrinsic difference between the two cell types (Figure 7). CB-MSCs show a high upregulation of CEBPα and PPARγ after treatment with 5 µM etoposide on d0 of adipogenic differentiation, whereas BM-MSCs exhibit no significant change in all conditions. CEBPβ levels showed no significant changes under all treatment conditions (Figure S5).

- *Chondrogenic Differentiation*

**Figure 8:**
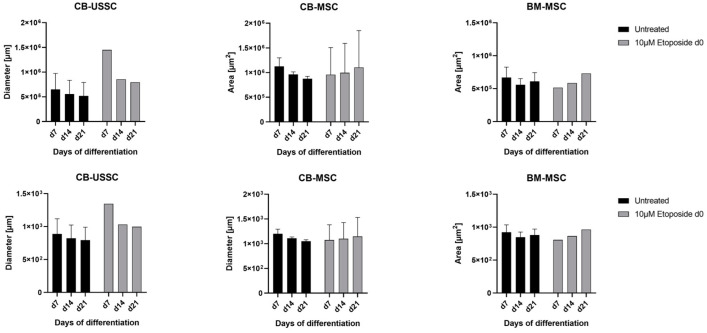
Chondrogenic differentiation of MSCs after 24 h etoposide treatment on d0 of differentiation. Areas and diameters of the chondrogenic pellets were measured on d7, d14, and d21 of chondrogenic differentiation (Mean + SD N = 3, n = 3). Abbreviations: BM, bone marrow; CB, cord blood; d; day; MNU, N-methyl-N-nitroso-urea; USSC; unrestricted somatic stem cells.

A total of 10 µM etoposide treatment on d7 of chondrogenic differentiation did not affect pellet condensation of CB-USSC and BM-MSC but led to an increase in the diameter and area of chondrogenic pellets derived from CB-MSCs (Figure 8).

#### 3.3.3. Effects of MNU Treatment on Day 7 of Differentiation

- *Osteogenic Differentiation*

**Figure 9:**
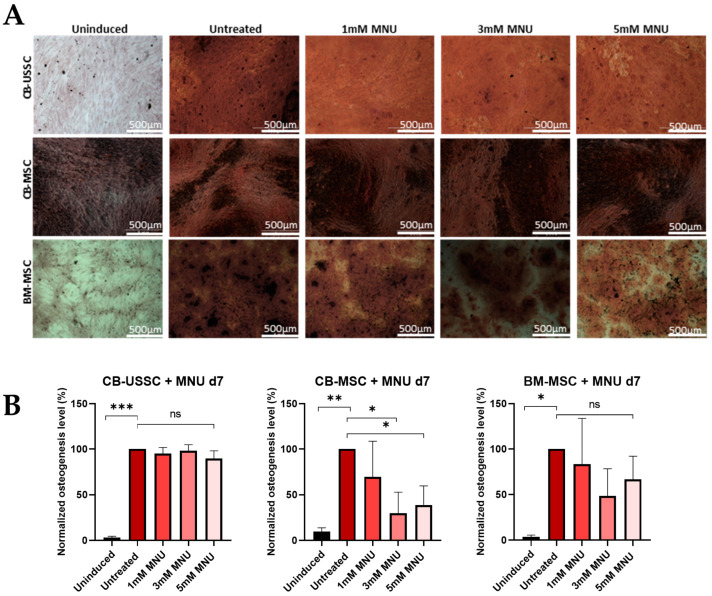
(**A**) Osteogenic differentiation of MSCs after 1 h MNU treatment on d7. Representative data (CB-USSC4-CB-MSC4-BM-MSC4) after Alizarin Red S staining on d14 of osteogenic differentiation is shown. Scale bars = 500 µm. (**B**) Osteogenic differentiation of MSCs after 1 h MNU treatment with different doses (untreated, 1 mM, 3 mM, and 5 mM) on d7. Quantitative determination of Alizarin Red S staining on d14 of osteogenic differentiation is shown. Quantitative data are presented as means ± SD of N = 3, n = 3. Differences were considered significant at *p* ≤ 0.05. * denotes *p* ≤ 0.05, ** denotes *p* ≤ 0.01, *** denotes *p* ≤ 0.001 and ns denotes non-significant. Abbreviations: BM, bone marrow; CB, cord blood; d, day; MNU, N-methyl-N-nitroso-urea; USSC, unrestricted somatic stem cells.

MSC treatment with MNU on d7 after induction of osteogenic differentiation had no significant effect on osteogenesis of CB-USSCs and BM-MSCs but led to a significant decrease in calcification levels of CB-MSCs (Figure 9).

RUNX2 levels did not show any significant dose-dependent changes upon MNU treatment on d7. In contrast, OSX was downregulated in a dose-dependent manner in CB-USSCs and CB-MSCs. BM-MSCs showed increasing OSX levels with increasing genotoxin dose (Figure S6).

- *Adipogenic Differentiation*

**Figure 10:**
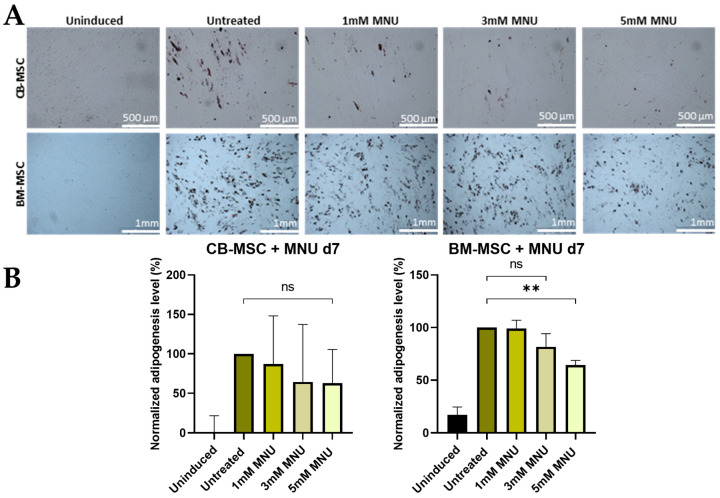
(**A**) Adipogenic differentiation of MSCs after 1 h MNU treatment on d7. Representative data (CB-MSC3-BM-MSC1) after Oil Red O staining on d21 of adipogenic differentiation is shown. The induced samples successfully underwent adipogenesis, while the uninduced sample was devoid of characteristic lipid droplet formation after 21 days of differentiation. Scale bars = 500 µm or 1 mm. (**B**) Adipogenic differentiation of MSCs after 1 h MNU treatment with different doses (untreated, 1 mM, 3 mM, and 5 mM) on d7. Quantitative determination of Oil Red O staining on d21 of adipogenic differentiation is shown. Quantitative data are presented as means ± SD of N = 3, n = 3. Differences were considered significant at *p* ≤ 0.05. ** denotes *p* ≤ 0.01, and ns denotes non-significant. Abbreviations: BM, bone marrow; CB, cord blood; d, day; MNU, N-methyl-N-nitroso-urea.

Treatment of CB-MSCs with MNU on d7 of adipogenic differentiation had no significant effect on their adipogenic potential. BM-MSCs showed a decreased adipogenic potential after MNU treatment of d7 only after treatment with the highest genotoxin dose. Here, again, CB-MSCs showed a reduced adipogenic differentiation potential compared with the adult BM-MSCs (Figure 10). No significant changes regarding CEBPβ and PPARγ gene expression were registered upon MNU treatment (Figure S7).

- *Chondrogenic Differentiation*

**Figure 11:**
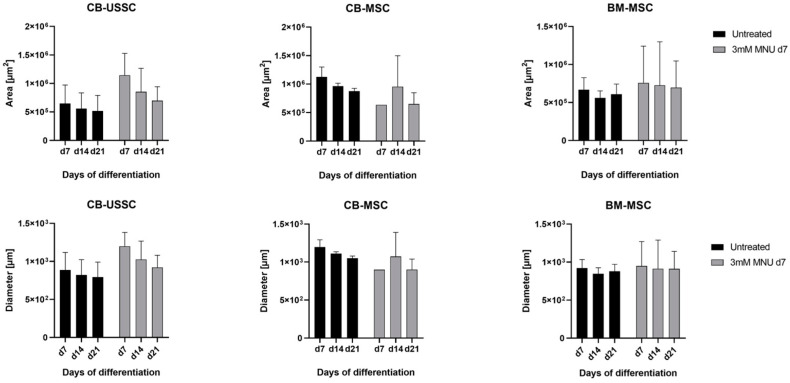
Chondrogenic differentiation of MSCs after 1 h MNU treatment on d7 of differentiation. Areas and diameters of the chondrogenic pellets were measured on d7, d14, and d21 of chondrogenic differentiation (Mean + SD N = 3, n = 3). Abbreviations: BM, bone marrow; CB, cord blood; d; day; MNU, N-methyl-N-nitroso-urea; USSC; unrestricted somatic stem cells.

Treatment of MSCs with 3 mM MNU on d7 of chondrogenic differentiation had no impact on CB-USSC but led to a condensation impairment in the case of CB-MSCs. The chondrogenic condensation of BM-MSCs was, in general, not as pronounced as in the neonatal MSCs (Figure 11).

#### 3.3.4. Effects of Etoposide Treatment on Day 7 of Differentiation

- *Osteogenic Differentiation*

**Figure 12:**
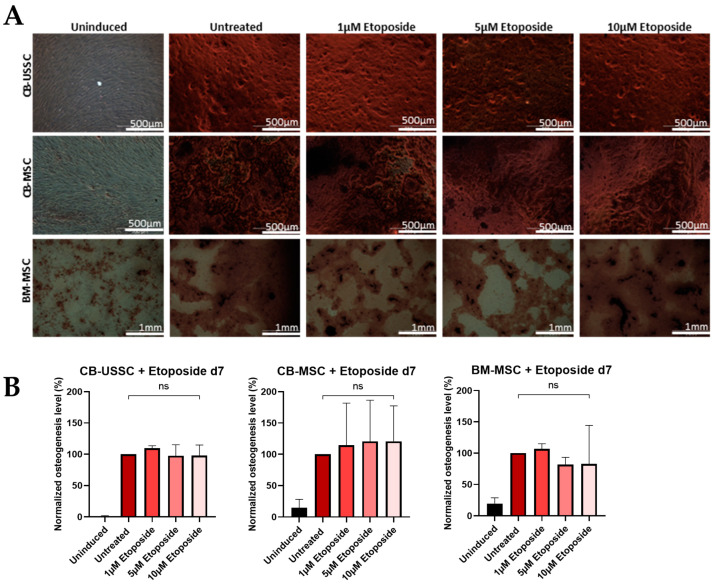
(**A**) Osteogenic differentiation of MSCs after 24 h etoposide treatment on d7. Representative data (CB-USSC4-CB-MSC3-BM-MSC6) after Alizarin Red S staining on d14 of osteogenic differentiation is shown. Scale bars = 1 mm. (**B**) Osteogenic differentiation of MSCs after 24 h etoposide treatment with different doses (untreated, 1 µM, 5 µM, and 10 µM) on d7. Quantitative determination of Alizarin Red S staining on d14 of osteogenic differentiation is shown. Quantitative data are presented as means ± SD of N = 3, n = 3. Differences were considered significant at *p* ≤ 0.05. ns denotes non-significant. Abbreviations: BM, bone marrow; CB, cord blood; d, day; USSC, unrestricted somatic stem cells.

Etoposide treatment of all MSC cell types on d7 of osteogenic differentiation had no effect on their ability to differentiate into osteocytes (Figure 12).

RUNX2 showed a genotoxic damage-dependent downregulation in CB-MSCs and BM-MSCs, whereas OSX levels were not significantly dysregulated in CB-USSCs and BM-MSCs, although a slight increase was registered in CB-MSCs (Figure S8).

- *Adipogenic Differentiation*

**Figure 13:**
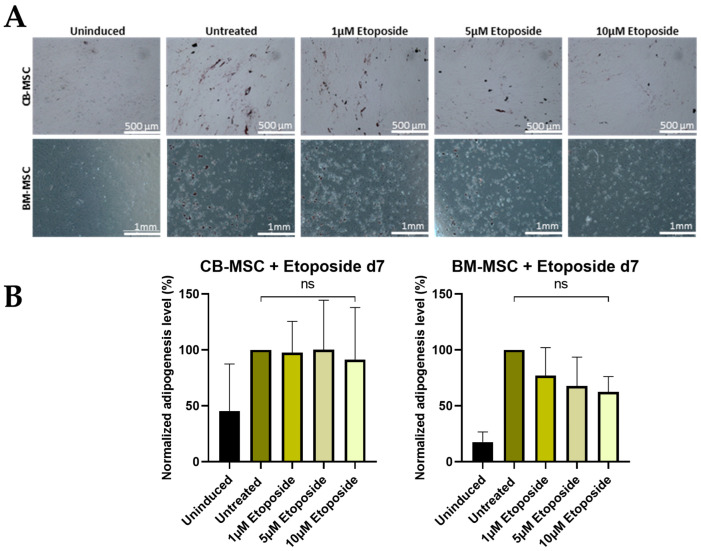
Adipogenic differentiation of MSCs after 24 h etoposide treatment on d7. (**A**) Representative data (CB-MSC3-BM-MSC2) after Oil Red O staining on d21 of adipogenic differentiation is shown. The induced samples successfully underwent adipogenesis, while the uninduced sample was devoid of characteristic lipid droplet formation after 21 days of differentiation. Scale bars = 500 µm or 1 mm. (**B**) Adipogenic differentiation of MSCs after 24 h etoposide treatment with different doses (untreated, 1 µM, 5 µM, and 10 µM) on d7. Quantitative determination of Oil Red O staining on d21 of adipogenic differentiation is shown. Quantitative data are presented as means ± SD of N = 3, n = 3. ns denotes non-significant. Abbreviations: BM, bone marrow; CB, cord blood; d, day.

Etoposide treatment of all MSC types on d7 had no effect on the outcome of adipogenic differentiation (Figure 13). CEBPα showed no differences between treated and untreated samples (Figure S9). CEBPβ levels on d21 showed no significant changes in BM-MSCs, whereas an upregulation was registered on d21 in CB-MSCs in a dose-dependent manner. PPARγ levels were slightly increased in CB-MSCs with increasing genotoxic dose, whereas no significant change was seen in BM-MSCs.

- *Chondrogenic Differentiation*

**Figure 14:**
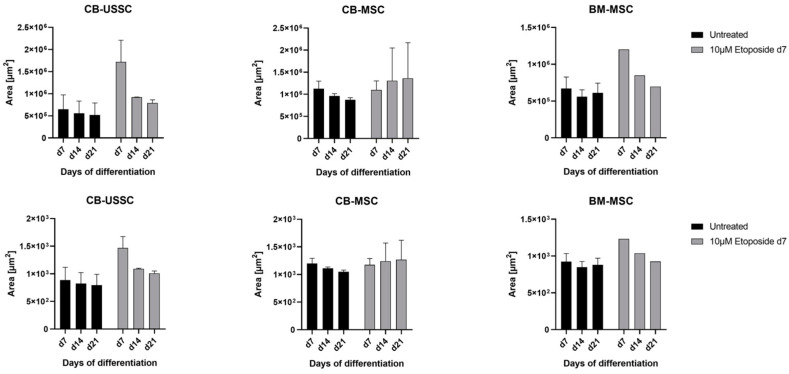
Chondrogenic differentiation of MSCs after 24 h etoposide treatment on d7 of differentiation. Areas and diameters of the chondrogenic pellets were measured on d7, d14, and d21 of chondrogenic differentiation (Mean + SD N = 3, n = 3). Abbreviations: BM, bone marrow; CB, cord blood; d, day; USSC, unrestricted somatic stem cell.

The results indicate that treatment with 10 µM etoposide on d7 of differentiation affects CB-USSCs, CB-MSCs, and BM-MSCs differently. While treated CB-USSCs and BM-MSCs showed an even higher condensation than the untreated control, CB-MSCs showed increasing pellet size over the differentiation period after etoposide treatment (Figure 14). This suggests that CB-MSCs may have a unique response to etoposide treatment, potentially reflecting a differential sensitivity or the presence of adaptation mechanisms in CB-MSCs as compared with CB-USSCs and BM-MSCs.

## 4. Discussion

In order to maintain their genomic integrity, cells developed adequate responses to DNA damage. This is of particular interest in the context of cell differentiation. For this, MSCs are an ideal in vitro model for studying genotoxin-induced stress responses due to their multipotency and the possibility of testing cells of different biological ages.

Our results show that iPSCs, which were used as controls for their embryonic similar age, exhibit a higher sensitivity towards the genotoxic agents MNU and etoposide compared with MSCs. This increased sensitivity could be due to more active cell division compared with MSCs and a lower DSB repair capacity of iPSCs. Others have reported that in spite of fast DNA damage repair capacity, iPSCs are more prone to apoptosis [24]. Furthermore, their DNA repair capacities are more heterogeneous depending on their source as compared with progenitor cells [24]. Moreover, the reprogramming process of iPSCs imposes oxidative stress and other cellular changes that can diminish their overall DNA repair efficiency [25].

In contrast to iPSCs, all types of MSCs were more resistant to the DNA-damaging agents MNU and etoposide. Due to their prolonged lifespan, they usually acquire high amounts of damage and may have specific damage coping mechanisms in order to avoid loss of functional activity. Others have shown that this resistance is associated with an induction of p53, proliferation arrest, or temporary G_2_/M cell cycle arrest upon genotoxic damage [26]. Despite the lower sensitivity of MSCs to damage, high MNU or etoposide concentrations led to significant morphological changes, indicating functional changes and impaired cell proliferation, regardless of the biological age, thus underlining the significant effect of genotoxic substances on cell integrity. Genotoxin treatment additionally led to decreased proliferation kinetics, which was more pronounced in adult BM-MSCs, even though this effect was not seen after analysis by comet assay. This is partly due to their lower basal proliferation activity compared with neonatal MSCs but might also be reinforced by the prior accumulated damage burden and the age-related decrease in their DNA repair capacity during the aging process.

The multilineage potential of MSCs is a valuable parameter that can be used for testing drug effects on cell differentiation. This parameter, however, has not been previously studied. Genotoxin treatment on d0 prior to the induction of osteogenic differentiation showed more severe effects compared with genotoxin treatment on d7, with the exception of etoposide-treated CB-MSCs. No significant changes were registered in the mRNA expression of RUNX2 and OSX, although others have described RUNX2 involvement in DDR by regulation of H2AX phosphorylation and its accumulation after genotoxic stress in muscular smooth muscle cells, thus leading to vascular calcification [27].

Genotoxin treatment applied on d0, prior to the induction of adipogenic and chondrogenic differentiation, did not significantly influence the differentiation outcomes in these lineages, unlike in osteogenic differentiation, which appears to be more susceptible to DNA damage at early time points of the differentiation process. This finding suggests that adipogenic and chondrogenic pathways may have greater resilience to genotoxic stress applied at early time points of differentiation, potentially due to differences in lineage-specific DNA repair mechanisms or stress response pathways that protect these differentiation routes from early-stage damage. In accordance with this hypothesis, no significant decreased levels in the expression of the adipogenic genes CEBPα, CEBPβ, and PPARγ were assessed. An exception was observed within CB-MSCs, which displayed a higher sensitivity to genotoxin treatment compared with CB-USSCs and BM-MSCs, suggesting an intrinsic vulnerability that may be due to differences in DNA repair efficiency or stress response pathways. This enhanced sensitivity of CB-MSCs highlights the need to consider cell-source-specific and age-related responses when evaluating the impact of genotoxins on the differentiation potential of MSCs.

Interestingly, genotoxic noxae administered on d7 after induction of differentiation appear to exert a generally reduced impact on MSC differentiation potential compared with treatment on d0, suggesting that cells might have adapted protective mechanisms during the late stages of differentiation, e.g., transcription-coupled repair or nucleotide excision repair [28]. Among the conditions tested, only MNU treatment on d7 showed an effect on osteogenic differentiation, specifically in CB-MSCs, underscoring their unique sensitivity to DNA damage even at later stages of differentiation induction. For other MSC types (CB-USSCs and BM-MSCs), no significant changes were observed across all differentiation lineages following MNU or etoposide treatment on d7.

Regarding chondrogenic differentiation, genotoxic stress on d0 did not affect this lineage significantly, and the same held true for treatment on d7, further reinforcing the idea that the chondrogenic pathway may possess a greater inherent resilience to DNA damage compared with the osteogenic pathway. This observed resistance in chondrogenic differentiation could indicate either an enhanced capacity for DNA repair in this lineage or a differential reliance on DDR pathways, e.g., non-homologous end joining, which may mitigate the effects of genotoxic agents. The extracellular matrix (ECM) synthesized during chondrogenic differentiation may play a protective role against genotoxic agents when treatment is applied to d7 of differentiation. This matrix, rich in proteoglycans, collagen, and other structural proteins, creates a dense, supportive environment that may help shield the cells from harmful agents. The ECM could act as a physical barrier, limiting the penetration and diffusion of genotoxic compounds into the cells and thus reducing their effective concentration at the cellular level. Additionally, the matrix might interact with cellular signaling pathways, potentially modulating cell responses to DNA damage and enhancing their resilience during later stages of differentiation. This potentially protective effect of the ECM may partially explain the reduced impact of genotoxin treatment on chondrogenic differentiation compared with earlier treatments in other lineages.

Our findings contribute significantly to the understanding of MSC differentiation and its therapeutic potential by highlighting genotoxin effects on undifferentiated and differentiated MSCs. By analyzing these effects at various differentiation time points, we have demonstrated that MNU- and etoposide-induced damage can not only impair cell viability and proliferation but might also affect the ability of MSCs to progress through their differentiation pathway, which is a critical factor for their therapeutic application in tissue repair and regeneration. Moreover, we have provided evidence about the resilience of differentiated MSCs compared with their undifferentiated counterparts, showing that the timing of genotoxic stress influences treatment outcomes. This is important to consider in the context of drug testing and safety assessment, and a deeper understanding of the responses at different stages of differentiation allows the development of more refined in vitro models to better mimic physiological conditions.

Nevertheless, a limitation of this study is the relatively small sample size and the inherent variability in donor characteristics accentuating MSC-related heterogeneity. These factors may influence the observed responses among the different cell lines. While efforts were made to minimize these effects, individual donor variability, including genetic and epigenetic differences, could still contribute to variations in the results. Future studies with larger sample sizes and more standardized donor selection criteria will be essential to validate and generalize these findings.

## 5. Conclusions

The insights obtained from this study emphasize the critical need to consider the effects of genotoxic damage on cell differentiation in an agent- and biological age-specific manner when conducting toxicological studies and during drug development. Neonatal MSCs, with their enhanced proliferative capacity, differentiation potential, and lower immunogenicity, present a significant promise for regenerative therapies and are a valuable tool for new approach methodologies to avoid animal testing compared with adult MSCs. Furthermore, in addition to therapy-related hematotoxicity, adverse effects on MSCs, which are the main components of the hematopoietic stem cell niche, also need to be considered.

## Data Availability

The original contributions presented in the study are included in the article, further inquiries can be directed to the corresponding author.

## Supplementary Materials

The following supporting information can be downloaded at: https://www.mdpi.com/article/10.3390/cells13242134/s1, Table S1: List of used CB-USSC, CB-MSC and BM-MSC cell lines; Table S2: List of used primers for RT-qPCR; Figure S1: Expression pattern of DDR of an iPSC cell line (R26) compared to MSCs from different sources; Figure S2: Expression pattern of early and late osteogenic genes in CB-USSC, CB-MSC and BM-MSC on d7 or d14 after induction of osteogenic differentiation and 1 h treatment with MNU on d0 of differentiation; Figure S3: Expression pattern of late adipogenic genes in CB-MSC and BM-MSC on d21 after induction of adipogenic differentiation and 1 h treatment with MNU on d0 of differentiation; Figure S4: Expression pattern of early and late osteogenic genes in CB-USSC, CB-MSC and BM-MSC on d7 or d14 after induction of osteogenic differentiation and 24h treatment with etoposide on d0 of differentiation; Figure S5: Expression pattern of late adipogenic genes in CB-MSC and BM-MSC on d21 after induction of adipogenic differentiation and 24 h treatment with etoposide on d0 of differentiation; Figure S6: Expression pattern of early and late osteogenic genes in CB-USSC, CB-MSC and BM-MSC on d14 after induction of osteogenic differentiation and 1 h treatment with MNU on d7 of differentiation; Figure S7: Expression pattern of late adipogenic genes in CB-MSC and BM-MSC on d21 after induction of adipogenic differentiation and 1 h treatment with MNU on d7 of differentiation; Figure S8: Expression pattern of early and late osteogenic genes in CB-USSC, CB-MSC and BM-MSC on d14 after induction of osteogenic differentiation and 24 h treatment with etoposide on d7 of differentiation; Figure S9: Expression pattern of late adipogenic genes in CB-MSC and BM-MSC on d21 after induction of adipogenic differentiation and 1 h treatment with MNU on d7 of differentiation.
