# Supplementary material for: Evaluation of Genotoxic Effects of N-Methyl-N-Nitroso-Urea and Etoposide on the Differentiation Potential of MSCs from Umbilical Cord Blood and Bone Marrow
Source: Cells. 2024 Dec 23;13(24):2134. doi: 10.3390/cells13242134 (PMC11675027; doi:10.3390/cells13242134)
Supplement: Supplementary file 1 [file cells-13-02134-s001.zip › cells-3377307-supplementary.pdf]

Supplementary Materials:

Table S1: List of used CB-USSC, CB-MSC and BM-MSC cell lines, all derived from different donors and were numbered independently of the donor. The donor age of BM-MSCs is listed. Abbreviations: BM, bone marrow; CB, cord blood; d, day; USSC, unrestricted somatic stem cell; N.K., not known;

Cell type	Number	Name	Donor age
CB-USSC	1	SA5/73	Neonatal
	2	SA8/25	
	3	SA10/36	
	4	USSC86b	
CB-MSC	1	SA6/51	
	2	SA8/39	
	3	USSC63	
	4	USSC120a	
BM-MSC	1	KM9-14	23
	2	KM9-15	32
	3	KM114	43
	4	KM120	N.K.
	5	KM8/06	52
	6	KM1/23	36
	7	KM2/23	31
	8	KM3/23	24

Table S2: List of used primers for RT-qPCR. Abbreviations: ATM, Ataxia Telangiectasia Mutated; BRCA1, Breast Cancer 1; BRCA2, Breast Cancer 2; CEBP, CCAAT/Enhancer Binding Protein Alpha; O_6-Methylguanine-DNA Methyltransferase; NHEJ1, Non-Homologous End Joining Factor 1; OSX: Osterix; P21, Cyclin-Dependent Kinase Inhibitor 1A (CDKN1A); PPARγ, Peroxisome Proliferator-Activated Receptor Gamma; RAD51, RAD51 Recombinase; RPL13a, Ribosomal Protein L13a; RUNX2, Runt-Related Transcription Factor 2; TP53, Tumor Protein 53 (p53); XRCC: X-Ray Repair Cross-Complementing;

Target	Forward sequence 5´- 3´	Reverse sequence 5´- 3´
ATM	CCTTGTGCTAGTGGGCAGAA	ATGGGGAGCAAAGAACCCAG
BRCA1	CCACAGATCAACCTGGAATGG	GTAGAGTGCTACACTGCTCA
BRCA2	TTCTGAGGTGGACCTAATAGG	TGATTTGGATTCTGGTCGCC
CEBPα	GAGTCACACCAGAAAGCTAG	GATGGACTGATCGTGCTTC
CEBPβ	TTTCGAAGTTGATGCAATCG	ACAGCAACAAGCCCGTAGG
MGMT	ACCGTTTGCGACTTGGTACT	GGGCTGGTGGAAATAGGCAT
NHEJ1	CCATTGTTGAAGGACGCTGC	CTAGCTCCCTCACTTGGCAC
OSX	TGCTTGAGGAGGAAGTTCAC	CTGAAAGGTCACTGCCCAC
P21	TACATCTTCTGCCTTAGT	TCTTAGGAACCTCTCATT
PPARγ	TCCATGCTGTTATGGGTGAA	TCAAAGGAGTGGGAGTGGTC
RAD51	GCTGATGAGTTTGGTGTAGC	AACATAGCTTCAGCTTCAGG
RPL13a	GAGGTATGCTGCCCCACAAA	TTCAGACGCACGACCTTGAG
RUNX2	GAGTGGACGAGGCAAGAG	GGACACCTACTCTCATACTG
TP53	TTCCGAGAGCTGAATGAGGC	AATGTCAGTCTGAGTCAGGCC
XRCC4	CCTCTAGGAGAATCAGCTTCAAGA	AAAGAGGTCTTCTGGGCTGC
XRCC5	AGCATAGACTGCATCCGAGC	TCCCCATACATCCACGACCT
XRCC6	TGCGTGGATTGTCGTCTTCT	CTTCTTCATCGCCCTCGGTT

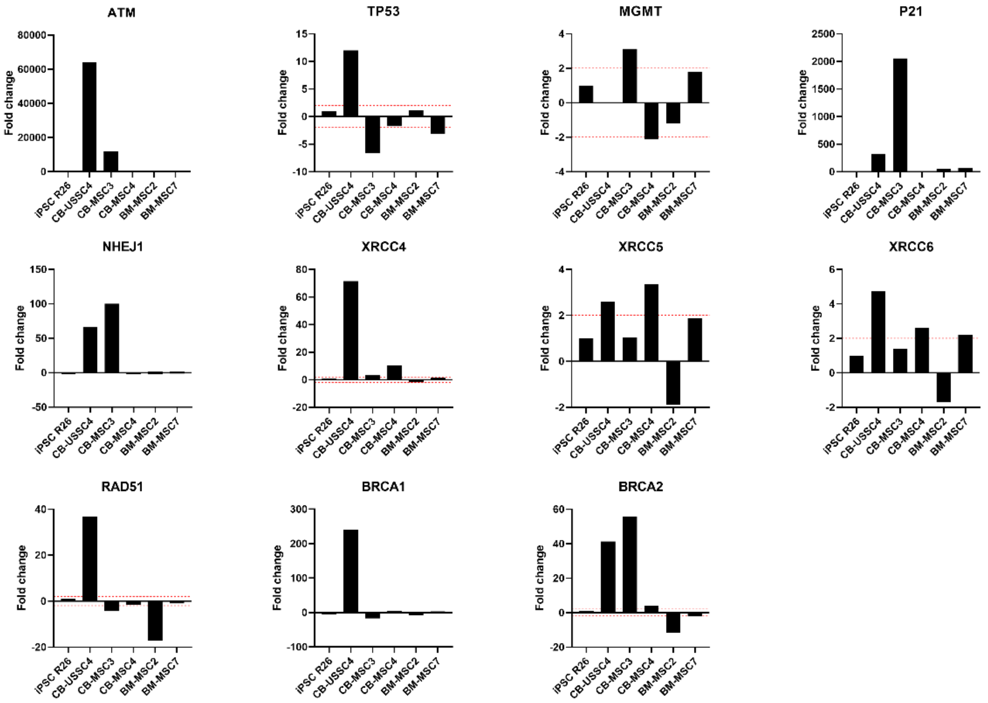

Figure S1: Expression pattern of DDR of an iPSC cell line (R26) compared to MSCs from different sources. Fold change was calculated using the $2^{-\Delta\Delta CT}$ method relative to the untreated control and normalized to the reference gene RPL13a. 2 represents a two-fold increase and -2 represents a two-fold decrease. Abbreviations: BM, bone marrow; CB, cord blood; iPSC, induced pluripotent stem cell; MSC, mesenchymal stromal cell; USSC, unrestricted somatic stem cell;

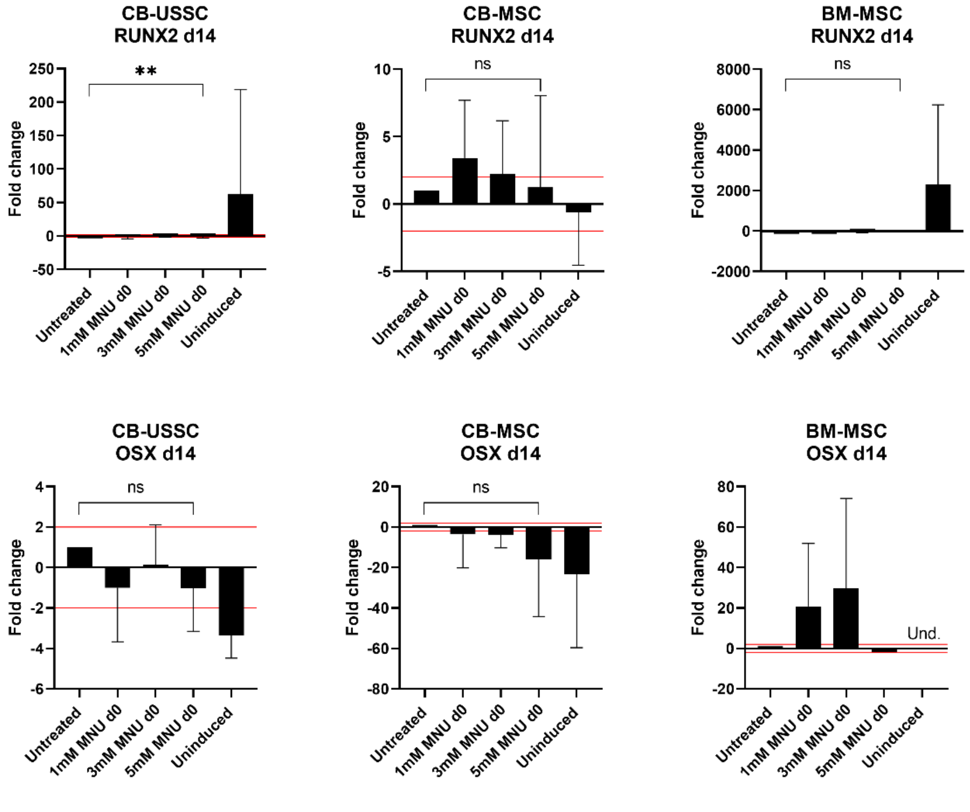

Figure S2: Expression pattern of early and late osteogenic genes in CB-USSC, CB-MSC and BM-MSC on d7 or d14 after induction of osteogenic differentiation and 1 h treatment with MNU on

d0. Fold change was calculated using the $2^{-\Delta\Delta CT}$ method relative to the untreated control and normalized to the reference gene RPL13a. The red lines indicate the significance thresholds. 2 represents a two-fold increase and -2 represents a two-fold decrease. Abbreviations: d, day; RUNX2, Runt-related transcription factor 2; OSX, Osterix; Und., undetermined;

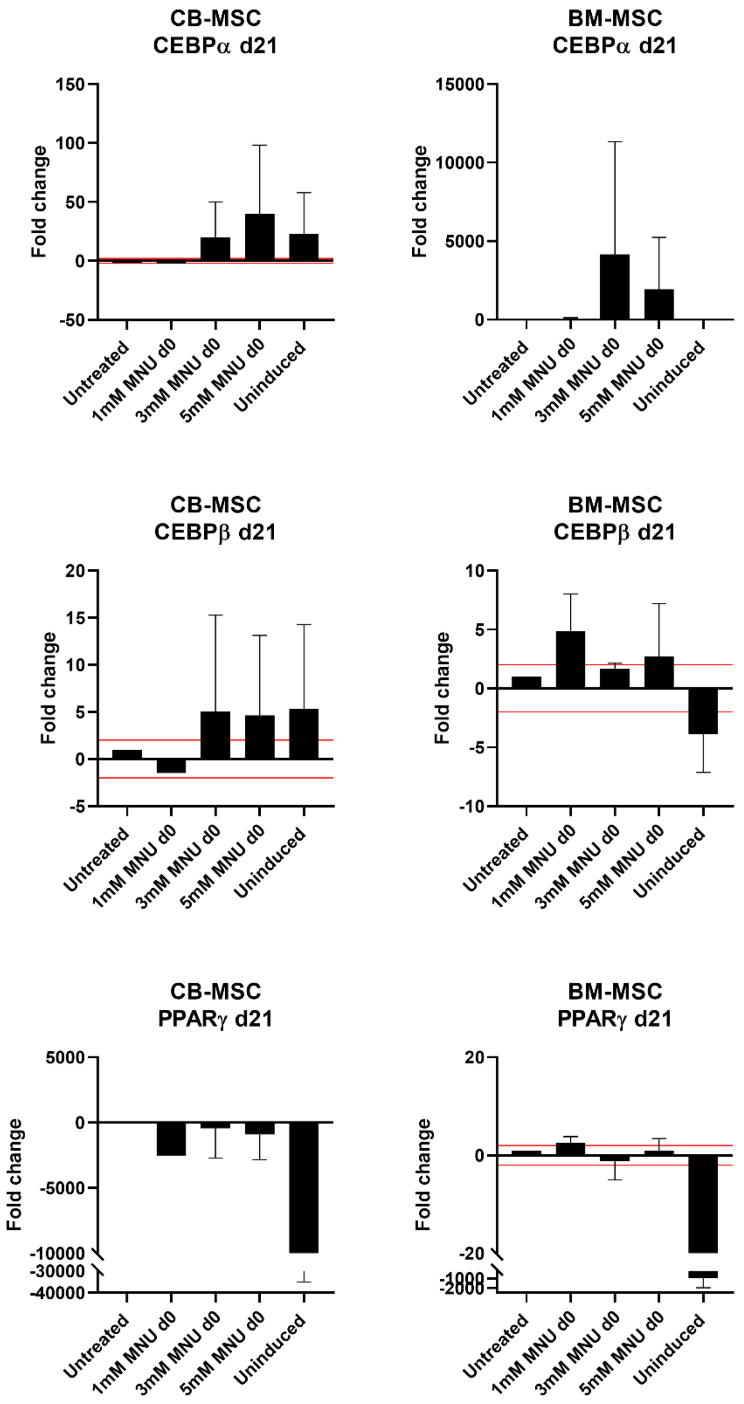

Figure S3. Expression pattern of late adipogenic genes in CB-MSC and BM-MSC on d21 after induction of adipogenic differentiation and 1 h treatment with MNU on d0. Fold change was calculated using the $2^{-\Delta\Delta CT}$ method relative to the untreated control and normalized to the reference gene RPL13a. The red lines indicate the significance thresholds. 2 represents a two-fold increase and -2 represents a two-fold decrease. Abbreviations: PPARγ, Peroxisome proliferator-activated receptor gamma; CEBP, CCAAT-enhancer-binding protein; d, day; MNU, N-methyl-N-nitroso-urea;

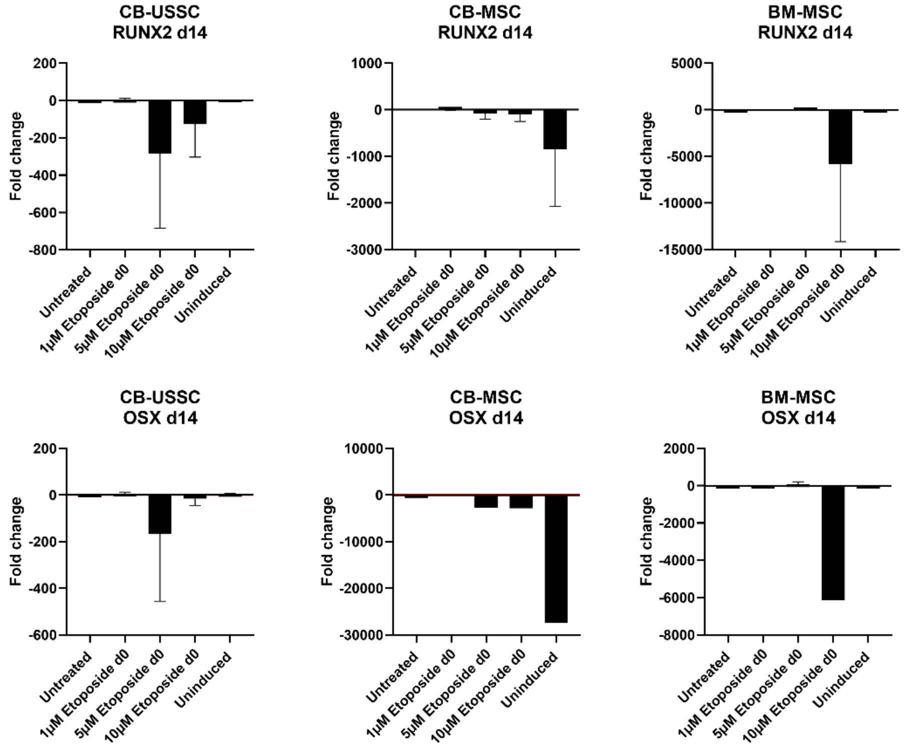

Figure S4. Expression pattern of early and late osteogenic genes in CB-USSC, CB-MSC and BM-MSC on d7 or d14 after induction of osteogenic differentiation and 24h treatment with etoposide. Fold change was calculated using the $2^{-\Delta\Delta CT}$ method relative to the untreated control and normalized to the reference gene RPL13a. The red lines indicate the significance thresholds. 2 represents a two-fold increase and -2 represents a two-fold decrease. Abbreviations: d, day; RUNX2, Runt-related transcription factor 2; OSX, Osterix;

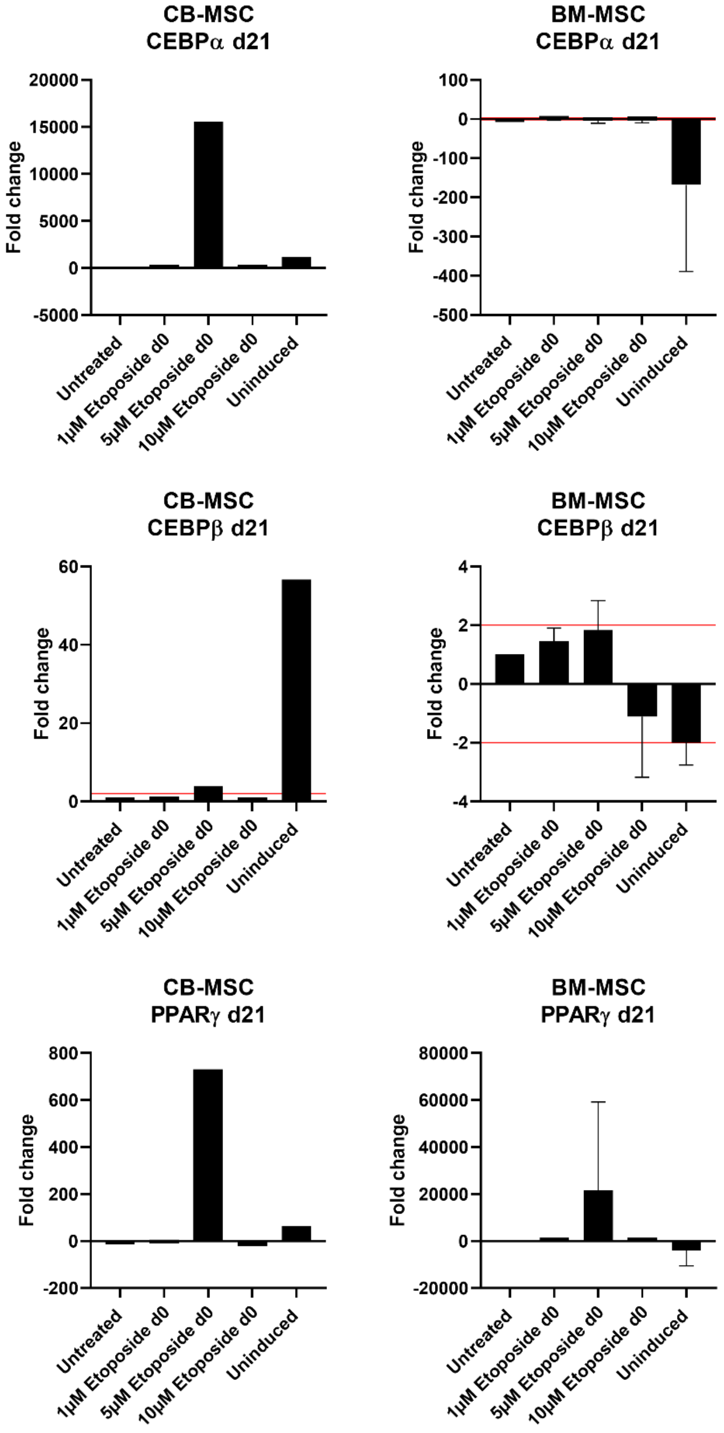

Figure S5. Expression pattern of late adipogenic genes in CB-MSC and BM-MSC on d21 after induction of adipogenic differentiation and 24 h treatment with etoposide on d0. Fold change was calculated using the $2^{-\Delta\Delta CT}$ method relative to the untreated control and normalized to the reference gene RPL13a. The red lines indicate the significance thresholds. 2 represents a two-fold increase and -2 represents a two-fold decrease. Abbreviations: PPARγ, Peroxisome proliferator-activated receptor gamma; CEBP, CCAAT-enhancer-binding protein; d, day;

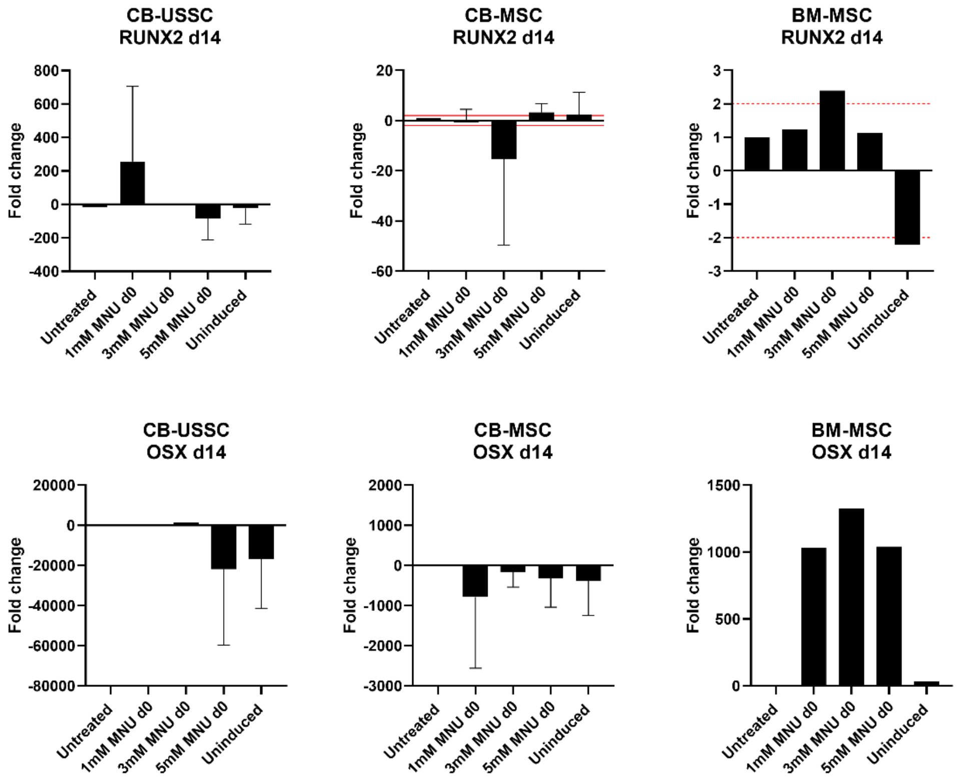

Figure S6. Expression pattern of early and late osteogenic genes in CB-USSC, CB-MSC and BM-MSC on d14 after induction of osteogenic differentiation and 1 h treatment with MNU on d7 of differentiation. Fold change was calculated using the $2^{-\Delta\Delta CT}$ method relative to the untreated control and normalized to the reference gene RPL13a. The red lines indicate the significance thresholds. 2 represents a two-fold increase and -2 represents a two-fold decrease. Abbreviations: d, day; RUNX2, Runt-related transcription factor 2; OSX, Osterix;

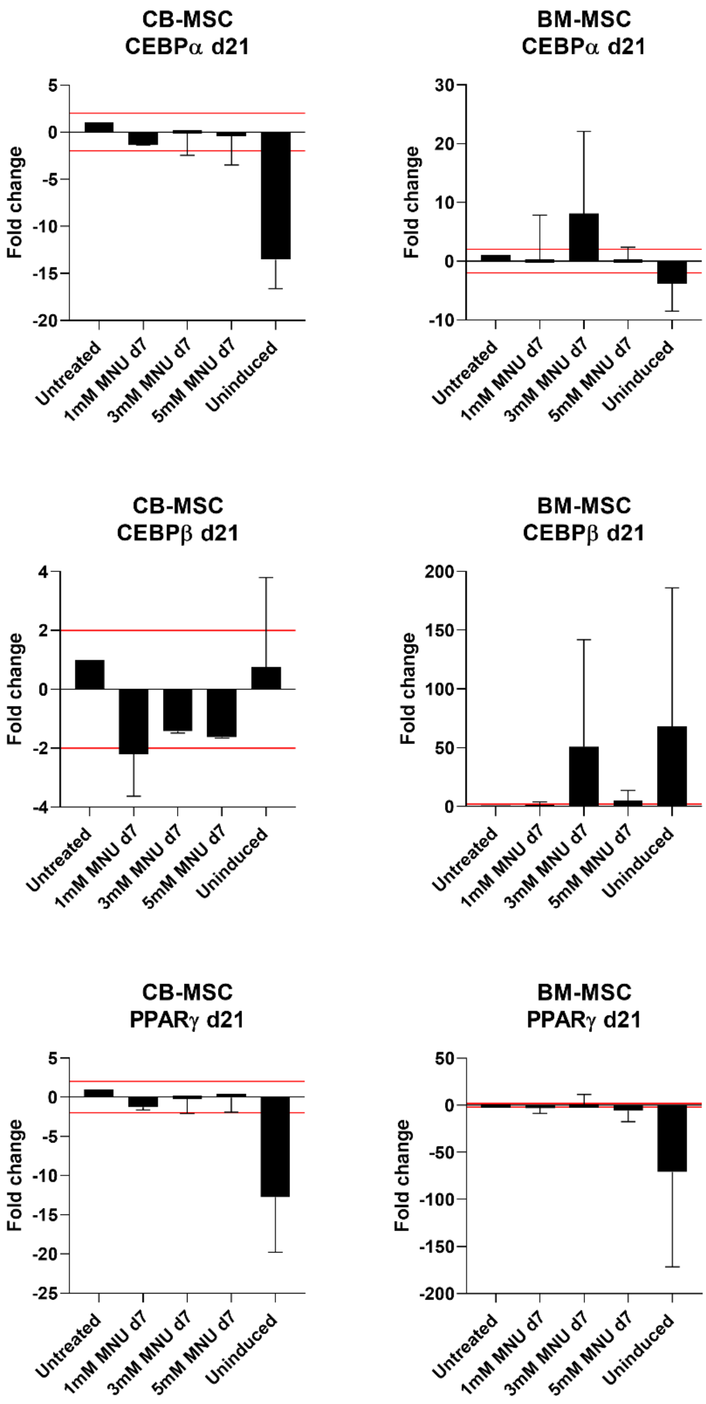

Figure S7. Expression pattern of late adipogenic genes in CB-MSC and BM-MSC on d21 after induction of adipogenic differentiation and 1 h treatment with MNU on d7. Fold change was calculated using the $2^{-\Delta\Delta CT}$ method relative to the untreated control and normalized to the reference gene RPL13a. The red lines indicate the significance thresholds. 2 represents a two-fold increase and -2 represents a two-fold decrease. Abbreviations: PPARγ, Peroxisome proliferator-activated receptor gamma; CEBP, CCAAT-enhancer-binding protein; d, day;

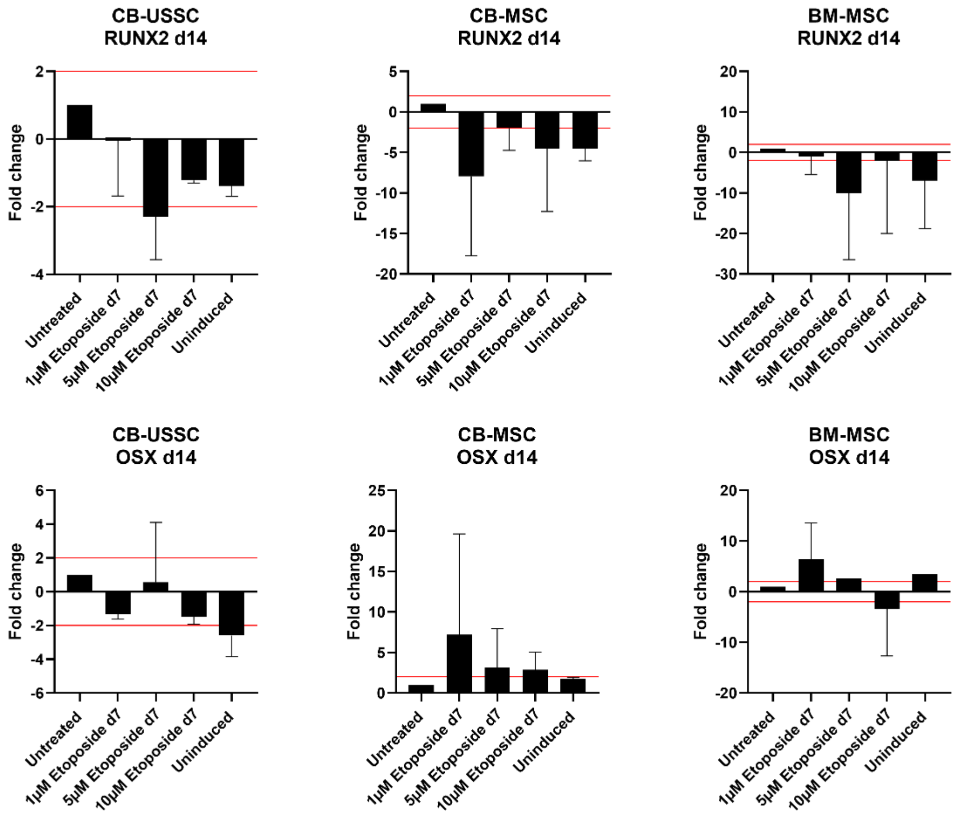

Figure S8. Expression pattern of early and late osteogenic genes in CB-USSC, CB-MSC and BM-MSC on d14 after induction of osteogenic differentiation and 24 h treatment with etoposide on d7 of differentiation. Fold change was calculated using the $2^{-\Delta\Delta CT}$ method relative to the untreated control and normalized to the reference gene RPL13a. The red lines indicate the significance thresholds. 2 represents a two-fold increase and -2 represents a two-fold decrease. Abbreviations: d, day; RUNX2, Runt-related transcription factor 2; OSX, Osterix;

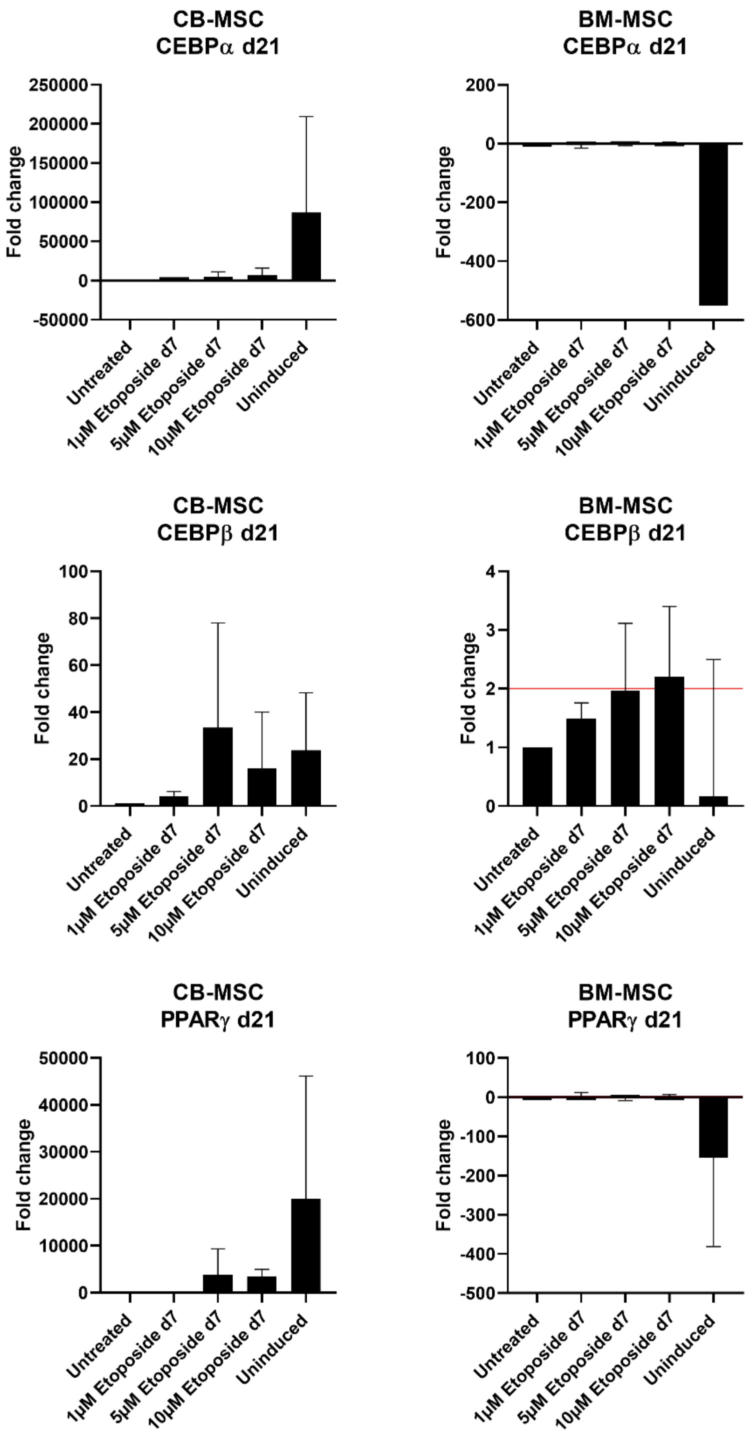

Figure S9. Expression pattern of late adipogenic genes in CB-MSC and BM-MSC on d21 after induction of adipogenic differentiation and 1 h treatment with MNU on d7. Fold change was calculated using the $2^{-\Delta\Delta CT}$ method relative to the untreated control and normalized to the reference gene RPL13a. The red lines indicate the significance thresholds. 2 represents a two-fold increase and -2 represents a two-fold decrease. Abbreviations: PPARγ, Peroxisome proliferator-activated receptor gamma; CEBP, CCAAT-enhancer-binding protein; d, day;
